# Language and Race


**Published:** 1894-06-30

**Authors:** 


### THE STUDY OF MAN.

### VII.—LANGUAGE AND RACE.*

ONE of the earliest definitions, or rather descriptions, of man, is that of "the talking animal"; and in its strictest and fullest sense, no doubt, language is his peculiar gift. But there is a language without words, a speech of gestures, and cries, fully intelligible to others as far as it goes, which, though possessed by man and merging in his formal language, is by no means confined to his use. The cries of animals, and of some birds in particular, have a variety and definiteness of meaning which cannot be wholly ascribed to our imaginative interpretation of them. The sounds of which they consist seem to be all representations of states of feeling, which very naturally arouse similar pleasures, anxieties and terrors in those who hear them, whether of the same species or not. The first speech of infant children, and the whole vocabulary of many imbeciles is composed of such inarticulate cries; and the language of some low types of savages seems at first hearing to consist of very little else: consonantal sounds being rare and obscure, and very slight variety of vocalisation being attempted.

It is a step of the first importance when child or animal begins to imitate the sounds which it hears in its neighbourhood. Perhaps this first occurs in the case of the emotional cries already mentioned, and has the effect of perfecting the code, so to speak, which regulates them; for the individual who misunderstands, or is misunderstood, on occasion of an alarm-cry, for instance, is at a very obvious disadvantage. It is very difficult to name an instance where an animal does more than this; some birds are said to mimic the cries of other species, and there are few doubtful cases of decoy cries used by predacious animals. Perhaps this "use of language to conceal thought" may be further illustrated by the feigned disablement and cries of pain of the lapwing to distract attention from the nest.

Now, it is just this power of imitating the sounds of other creatures and things which distinguishes the human infant from the young of all other animals. Not only the cries of animals, but the sounds of inanimate things, such as locomotives, machinery, bells, wind, rain, and thunder are represented, not always very accurately, but vividly enough, in such words as bow-wow, hee-haw; puff-puff, ding-dong; whirr, whistle, patter, thunder (German Donner: Romaic Boumbourià), which are recognisable at once; and many more which have lost their original significance, and now are used only metaphorically. Some twenty years ago the supporters of what became known as the "bow-wow" theory attempted to make this the main origin of all existing word-roots, and did much good work, even where their detailed applications of their methods were not convincing.

The second step is practically involved in the first, for no sooner does a child—or an adult, for that matter—coin an onomatopoieic word of this kind, than he proceeds to use it as a sign to indicate that he is thinking of the thing which it describes; or of one of its conspicuous qualities. The phrase "bow-wow—puff-puff" for instance, is a fairly intelligible combination of the ideas of "dog" and of "swift motion"; whether it means "run, doggie," or "the dog runs," or the running dog," is decided by the intonation of the speaker. Substantives and verbs; cases, moods, and tenses, are not yet distinguished, it is true; but neither are they in fully-developed Chinese, which has no grammar except the conventional rule that certain combinations of roots have this, and not that, of all possible meanings; and the really psychological rules of the "natural order of thought" which are common to all primitive spoken languages, as well as to the sign-language of the Redskins, and to the spontaneous sign-grammar of European deaf-mutes.

Intonation and gesture, however, become very soon so complicated that they are unintelligible at a short distance, or to a comparative stranger, as, for instance, in the different quarters of modern Naples; and it becomes necessary, in order to keep the meaning clear, to modify the principal signs, so as to indicate their relation to one another; epithets, for instance, are attached to the word to which they belong by some slight change, either at the beginning, as in East Africa; at the end, as in most European languages; or by insertion of a determinative syllable in the middle of their roots. Verbs—at first indistinguishable from substantives except by accent and position in the sentence—are distinguished by abbreviated forms of the signs for I, thou, he, and the like, and by other additions, limiting the action in time and otherwise. Basque, for instance, uses different grammatical forms according as the speaker approves or disapproves the agent, the action, or the object in question; while some Malay dialects solve the problem by using wholly different words, for instance, for striking with a thick stick or with a thin one; on an edge or a surface; and so on. Other shades of meaning are conveyed by prefixes and suffixes, or, as in Semitic languages, by alteration of the vowels of the root, *e.g.*, from the Arabic root q-t-l; killing, comes uqtul, to kill; iqtal, to cause killing; quatl, murder; and qitl, an enemy—all according to quite definite rules.

There are countless things and ideas, however, which cannot be imitated in sound; and it is the most difficult matter to determine how the existing expressions for them were arrived at. But it becomes probable that a very large majority arise by the extension of a sound sign to express some other thing which has a likeness in some particular with that which it properly represents. The Romaic for "warm," for instance, in Zestò (ζεστὸ) which simply means "boiling" in Old Greek, and is descriptive of the sound of boiling water; now it is not applied to *boiling*, for which a new word, brasto (βραστὸ) is commonly used. To pursue this subject, however, leads us far into the special domain of philology, and we must here confine ourselves to the points where that study throws light directly on the history and development of man.

It has been indicated already that languages build up their root-sounds into detailed statements of feelings or of external facts in various ways, which can be

* The handiest summary of both sides of the Aryan question is Canon Taylor's "Origin of the Aryans" (W. Scott, 1890); for the philological side at its best and amplest, Schroder's "Prehistoric Antiquities of the Aryan Peoples," English translation by F. B. Jevons. (Griffin), second edition, 1889, containing an admirable summary of the history of the controversy.

used to frame a classification of the principal types of speech. The modification of vowels, for example, occurs only as a regular grammatical system in a group of languages spoken almost exclusively by races of Semitic descent; and such exceptions as exist can be shown to be due to conquest or assimilation of other races by Semitic peoples, *e.g.*, Arabic-speaking negroes and other African races. Similarly the purely monosyllabic type, which has no grammatical modifications, but build their sentences out of word-roots, like a wall out of bricks, is most fully developed in China and its immediate neighbours, Burmah and Siam, where there is reason to believe in a community of race. The Toki-speaking and Yoruba-speaking peoples of West Africa similarly seem to form ethnological as well as linguistic groups; and instances of the kind may be multiplied to an indefinite extent.

In many cases, however, the evidence of linguistic and of morphological anthropology leads to very different conclusions, and much difficulty has been caused, over and over again, by insisting on identity of race on the ground of identity or similarity of language. A conspicuous example of the confusion thus created, which has done more than anything else to discredit the philology of the last generation, is the conclusion which was drawn, from the general correspondence, both in vocabulary and structure, of the principal languages of Europe with the Zend of Persia and the Sanskrit of India, to the ethnological kinship of at least the great mass of those who speak these tongues; quite regardless of the conspicuous differences in outward appearance, skeletal proportions, and habits of life which are observed to exist between them. This "Aryan Hypothesis" deserves a far fuller treatment than can be given to it in these limits; but the discussion of it is at present less valuable for the positive results which have been established hitherto, than as a particularly striking example from the elaboration with which it has been worked out, of the variety of evidence which has to be dealt with in the problems of human origins, and of the difficulty in assigning to each class of material its proper weight in the computation. The question, in fact, at issue really is, Is it in this or that case more probable that a people should have changed its physical features without radical change of language; or that it should have learnt a new language from a few energetic conquerors, whose blood became lost in that of the older and conquered inhabitants without producing any perceptible change in their features, complexion, or internal structure.

